# Using Reduced Inoculum Densities of* Mycobacterium tuberculosis* in MGIT Pyrazinamide Susceptibility Testing to Prevent False-Resistant Results and Improve Accuracy: A Multicenter Evaluation

**DOI:** 10.1155/2017/3748163

**Published:** 2017-11-08

**Authors:** Glenn P. Morlock, Frances C. Tyrrell, Dorothy Baynham, Vincent E. Escuyer, Nicole Green, Youngmi Kim, Patricia A. Longley-Olson, Nicole Parrish, Courtney Pennington, Desmond Tan, Brett Austin, James E. Posey

**Affiliations:** ^1^Division of Tuberculosis Elimination, Laboratory Branch, National Center for HIV/AIDS, Viral Hepatitis, STD, and TB Prevention, Centers for Disease Control and Prevention, 1600 Clifton Road NE, Atlanta, GA 30329, USA; ^2^Tennessee Department of Health, Division of Laboratory Services, 630 Hart Lane, Nashville, TN 37216, USA; ^3^Mycobacteriology Laboratory, Wadsworth Center, New York State Department of Health, 120 New Scotland Avenue, Albany, NY 12208, USA; ^4^Public Health Laboratory, Los Angeles County Department of Public Health, 12750 Erickson Avenue, Los Angeles, CA 90242, USA; ^5^Mycobacteriology Laboratory, Wisconsin State Laboratory of Hygiene, 465 Henry Mall, Madison, WI 53706, USA; ^6^Missouri State Public Health Laboratory, 101 North Chestnut Street, Jefferson City, MO 65102, USA; ^7^Department of Pathology, Johns Hopkins School of Medicine, 600 North Wolfe Street, Baltimore, MD 21287, USA; ^8^Bureau of Clinical Laboratories, Alabama Department of Public Health, 8140 AUM Drive, Montgomery, AL 36117, USA; ^9^Massachusetts State Public Health Laboratory, 305 South Street, Jamaica Plain, MA 02130, USA; ^10^County of San Diego Public Health Laboratory, 3851 Rosecrans Street, San Diego, CA 92110, USA

## Abstract

The primary platform used for pyrazinamide (PZA) susceptibility testing of* Mycobacterium tuberculosis* is the MGIT culture system (Becton Dickinson). Since false-resistant results have been associated with the use of this system, we conducted a multicenter evaluation to determine the effect of using a reduced cell density inoculum on the rate of false resistance. Two reduced inoculum densities were compared with that prescribed by the manufacturer (designated as “BD” method). The reduced inoculum methods (designated as “A” and “C”) were identical to the manufacturer's protocol in all aspects with the exception of the cell density of the inoculum. Twenty genetically and phenotypically characterized* M. tuberculosis* isolates were tested in duplicate by ten independent laboratories using the three inoculum methods. False-resistant results declined from 21.1% using the standard “BD” method to 5.7% using the intermediate (“A”) inoculum and further declined to 2.8% using the most dilute (“C”) inoculum method. The percentages of the resistant results that were false-resistant declined from 55.2% for the “BD” test to 28.8% and 16.0% for the “A” and “C” tests, respectively. These results represent compelling evidence that the occurrence of false-resistant MGIT PZA susceptibility test results can be mitigated through the use of reduced inoculum densities.

## 1. Introduction

The antituberculous property of pyrazinamide (PZA) was first reported in 1952 [1] and the results of the first animal studies [2, 3] and human clinical trial [4] were published in that same year. In a phenomenon that has been termed the “paradox of PZA,” the drug has little* in vitro* activity against* M. tuberculosis* under standard culture conditions, yet it has high* in vivo* activity [5]. This paradoxical behavior is explained by the necessity of an acidic milieu for PZA activity [6]. PZA plays a critical role in tuberculosis treatment regimens due to its sterilizing activity against semidormant bacilli sequestered within the acidic environment of the macrophage [7, 8]. This unique property of PZA facilitated the shortening of current tuberculosis (TB) treatment regimens and PZA continues to be a standard component in clinical drug combination trials, including those featuring the newly approved drugs bedaquiline and delamanid [9].

PZA is a prodrug requiring pyrazinamidase (PZase) mediated conversion to pyrazinoic acid (POA). The physiological role of PZase is to convert nicotinamide to nicotinic acid as part of the bacterial NAD salvage pathway [10]. When nicotinamide is the substrate, the enzyme is designated as nicotinamidase. PZA can serve as an alternate substrate due to its close structural similarity to nicotinamide. Both reactions result in the deamination of substrates to their respective carboxylic acids, producing ammonia as a byproduct. PZase is encoded by the gene* pncA* [11]. Since the efficacy of PZA is contingent upon PZase catalyzed conversion of PZA to POA, a* pncA* mutation that can eliminate, or diminish, this enzymatic activity is predicted to confer PZA resistance. DNA sequencing to detect* pncA* mutations has been shown to be highly correlative with PZA resistance. Two meta-analyses reported overall sensitivities of 83% and 87% for* pncA* sequencing [12, 13]. In practice, DNA sequencing of* pncA* has been proven to be a powerful tool for genotypic diagnosis of PZA resistance in* M. tuberculosis*. Two significant caveats to this approach are that it cannot be assumed a priori that all mutations confer resistance and, conversely, that the lack of a mutation indicates susceptibility. The impact of a specific* pncA* mutation must be established either through functional genetics or through repetitious association with a resistant phenotype. Mutations that do not confer resistance have been reported [14, 15]. PZA resistance in isolates with wild-type* pncA* has been observed and is attributed to other mechanisms [16–18]. Due to these limitations, it remains necessary to perform culture-based PZA susceptibility testing.

Currently, there are two FDA-cleared platforms for PZA susceptibility testing; the BACTEC™ MGIT™ (Becton Dickinson (“BD”), Sparks, MD) system (henceforth designated as “MGIT system”) and the Versa-TREK MYCO TB™ (TREK Diagnostic Systems, Cleveland, OH) system. The MGIT system is the more commonly used of these two methods [19]. The use of the MGIT system has been associated with false-resistant results. In one report, 57 (7.7%) of 743 isolates were PZA-resistant when tested using MGIT, but when those isolates were tested using an alternate method, the radiometric BACTEC 460TB, only 33 (4.4% of total) were resistant. The remaining 24 (3.2%) isolates were susceptible; therefore, 42% of the MGIT resistant results were false-resistant [20]. False resistance using the MGIT system has also been observed in several proficiency testing surveys. A study conducted by the Swedish Institute for Communicable Disease Control found that, among two of five experienced clinical laboratories, the positive predictive values of resistant PZA MGIT results were 45% and 63% [21]. MGIT false resistance was also observed in the Model Performance Evaluation Program (MPEP), a proficiency evaluation survey administered by the U.S. Centers for Disease Control and Prevention (CDC). Over the course of seven surveys (35 test isolates) in 2012 through 2015, three isolates with no mutation in* pncA* repeatedly had a high rate of false-resistant results (22%, 78%, and 62%) [22–24]. Alternate PZA resistance associated genes such as* panD* and* rpsA* were not examined in these isolates. These reports, and others [20, 25], provide compelling evidence that PZA susceptibility testing using the MGIT system can produce false-resistant results.

Susceptibility testing of PZA requires using acidified culture media; the pH must be sufficiently low to allow for good PZA activity while not being so low as to inhibit bacterial growth solely on the basis of acidity. A pH of 5.5 to 6.0 has been determined to represent the best compromise between those two conditions [6, 26]. The requirement for an acidic environment for PZA activity has made susceptibility testing technically challenging. As the* in vitro* activity of PZA is highly dependent upon the acidity of the media, the neutralization of the media by the ammonia generated as a byproduct of the enzymatic conversion of PZA to POA is likely to affect PZase activity. An increase in pH from 5.5 to 6.1 is predicted to raise the PZA MIC from 50 to 200 *μ*g/ml [27]. Inoculum concentration is a critical factor affecting PZA activity [5]; the magnitude of the media pH increase is proportional to the number of viable bacilli used to inoculate the culture, and inoculation with a higher concentration of cells produces false resistance. Since the accuracy of PZA susceptibility testing can be contingent upon inoculum concentration, it is imperative to establish an inoculum cell density range that limits pH increase. The primary objective of this study was to determine whether the use of an inoculum cell density less than that prescribed in the MGIT package insert would mitigate the occurrence of false-resistant PZA susceptibility test results.

## 2. Materials and Methods

### 2.1. Study Participants

Ten laboratories participated in the study: six state and two county public health laboratories, a clinical laboratory, and the CDC Division of Tuberculosis Elimination Laboratory Branch (LB). The study was administered through funding provided by the CDC to the Association of Public Health Laboratories (APHL). Excluding the CDC laboratory, all laboratories were selected by a competitive process. Applications were reviewed by both CDC and APHL staff. Criteria evaluated in the scoring process included overall experience with MGIT PZA susceptibility testing, testing volume, and staff experience. The nine highest scoring laboratories were awarded a grant to conduct the required testing.

### 2.2. Strain Selection

The 15 strains of* M. tuberculosis* used in this study were obtained from a culture collection maintained by the CDC. Strains were phenotypically and genotypically well characterized. PZA MICs and* pncA* sequences of these strains (Table 1) were used to place isolates into three general groups: the first group that was comprised of strains that were always, or predominately (greater than 70% of test results), resistant to the PZA critical concentration of 100 *μ*g/ml using the MGIT system; the second group that was always, or predominately, PZA-susceptible; and the third group whose results vacillated between susceptible and resistant and were therefore classified as inconclusive.

### 2.3. Strain Preparation

Each of the 15 study strains was cultured in 100 ml of 7H9 Middlebrook broth supplemented with bovine albumin-dextrose-catalase at 37°C with occasional gentle agitation until turbid and then dispensed in 1 ml aliquots. Two vials were prepared for five of the strains. Duplicates had different identifiers and were included to evaluate reproducibility. In total, the study panel included 20 isolates. Vials were stored at −70°C until packaged and shipped to the study sites.

### 2.4. PZA Susceptibility Testing

**Figure 1:**
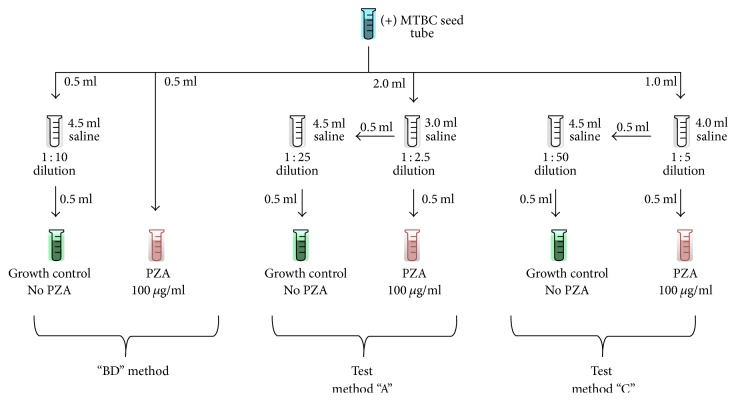
Procedural diagram of three MGIT PZA testing protocols.

The 10 laboratories performed PZA susceptibility testing following 3 different protocols using the BACTEC MGIT system (Figure 1). The first protocol was a minor modification of the manufacturer's protocol as described in the MGIT PZA kit package insert [28] and summarized here. Inocula were prepared by vortex-mixing a seed tube for 10 seconds and allowing it to settle for 15 minutes. A 0.5 ml aliquot was taken from the top of the settled seed tube and used to inoculate the culture tube containing 100 *μ*g/ml PZA (note: this method of inoculum preparation differs slightly from that described in the package insert which does not specify vortex-mixing the seed tube, allowing it to settle and aspirating from the top of the tube). Similarly, a second 0.5 ml aliquot was transferred to a tube containing 4.5 ml of sterile physiological saline yielding a 1 : 10 dilution. This dilution tube was then vortexed for 5 seconds and allowed to set for 5 minutes. A 0.5 ml aliquot taken from the top of this dilution tube was used to inoculate the tube without PZA (control). Each pair of culture tubes (control and PZA) was incubated in the MGIT instrument. Since this testing was performed following the manufacturer's instructions, we designated this as the “BD” method.

Each strain was tested using two additional protocols which we designated as methods “A” and “C.” These two methods were identical to the “BD” method with the exception of the seed tube dilutions used to inoculate the culture tubes. All dilutions were prepared in sterile saline. In method “A,” the control (no PZA) tube was inoculated with a 1 : 25 dilution of the seed tube and the PZA containing tube was inoculated with a 1 : 2.5 dilution of the seed tube. Method “C” used 1 : 50 and 1 : 5 seed tube dilutions, respectively, to inoculate the control and PZA tubes. It is important to emphasize that while the dilutions used in each of the three methods differed, the 1 : 10 ratio between control and PZA tubes was maintained (i.e., no dilution and 1 : 10 [“BD” method], 1 : 2.5 and 1 : 25 [“A” method], and 1 : 5 and 1 : 50 [“C” method]). In addition, the volume of inoculum was identical among all tubes.

### 2.5. Algorithm for Assigning Categorical PZA Susceptibility Status as the Predicted Result

An algorithm was developed to assign isolates to predicted categories of susceptible, resistant, or inconclusive (Table 1). This algorithm consisted of a progression of questions related to the* pncA* genotype and MGIT PZA result of each isolate. Eleven isolates lacked* pncA* mutations and were classified as susceptible. One isolate had a silent mutation and was accordingly classified as susceptible. MGIT PZA test results were used to classify the eight isolates with nonsynonymous or regulatory* pncA* mutations. Two* pncA* mutants (isolates 18 and 22) were always susceptible and two (isolates 4 and 14) were always resistant at 100 *μ*g/ml PZA. The classification of the remaining four isolates presented the greatest challenge because there was inconsistency among previous PZA susceptibility test results. One isolate (number 17) was classified as resistant because the results from previous PZA susceptibility tests were predominantly resistant. The previous PZA results for the other three isolates (numbers 19, 21, and 27) were highly variable and therefore were classified as inconclusive and not assigned a predicted categorical result.

### 2.6. Statistical Analysis

Results and dates of PZA testing were collected and entered into the provided Excel spreadsheets by participating laboratories. Once data collection was completed at each site, data were transmitted to the principal investigator. Analytic tools used for the evaluation of data included Microsoft Excel (Microsoft, Redmond, WA) and SPSS Version 21.0 (IBM Corp., Armonk, NY). Sensitivity, specificity, and positive and negative predictive values of MGIT for PZA testing were determined through cross-tabulation of results, with the predicted PZA susceptibility determination as a comparator. This study was determined to not be human subjects research by the U.S. CDC, National Center for HIV/AIDS, Viral Hepatitis, STD, and TB Prevention, as defined by 45 CFR 46.

## 3. Results

Data from one laboratory were excluded as the overall reproducibility of their duplicate tests was below 70%. Therefore, data for only 9 of the 10 participating laboratories are included in this analysis. Categorical PZA susceptibility test results from participating sites were compared to our algorithmically assigned predicted results. Since only resistant or susceptible study results were possible, there were four comparative outcomes: true-susceptible, false-susceptible, true-resistant, and false-resistant. True comparisons were those where the study result and predicted result were the same and false comparisons were those where the study result differed from the predicted result. Two strains (isolate number 19 and isolates 21 and 27) were considered “inconclusive” and not assigned a predicted result. Compiled MGIT PZA susceptibility test results are shown in Table 2. The number of valid tests shown in Table 2 differs from the expected total (306) because in some instances the test was not completed (“Timed Out”) within the 21-day instrument protocol. The percentage of true-susceptible results increased from 61.2% (186 of 304) using the standard (“BD”) method to 76.4% (226 of 296) using the “A” method (1 : 2.5 dilution) and to 79.2% (230 of 289) using the “C” method (1 : 5 dilution). The percentage of true-resistant results decreased from 17.1% (52 of 304) for the standard method to 14.2% (42 of 296) and 14.5% (42 of 289) for the “A” and “C” methods, respectively. The decline in true-resistant results with increasing inoculum dilution was accompanied by a corresponding increase in false-susceptible results. Notably, 81.8% (18 of 22) of these false-susceptible results involved one isolate (number 17) with the remaining four instances in another isolate (number 14) and all in the same laboratory. The “BD,” “A,” and “C” inoculum protocol results were 78.3% (238 of 304), 90.5% (268 of 296), and 94.1% (272 of 289) concordant, respectively, with the predicted results. False-resistant results declined nearly fourfold from 21.1% (64 of 304) using the standard “BD” method to 5.7% (17 of 296) using the intermediate (“A”) inoculum and to 2.8% (8 of 289) using the most dilute (“C”) inoculum method. Two false-susceptible results occurred using the “BD” method, while there were 11 and 9 instances using the “A” and “C” methods, respectively. Results obtained using the “BD,” “A,” and “C” inoculum protocols were 21.7% (66 of 304), 9.5% (28 of 296), and 5.9% (17 of 289) discordant, respectively, with the predicted results. Overall, substantially fewer false-resistant results occurred using the more dilute inocula methods (“A” and “C”) as compared to the standard (“BD”) inoculum. Study results for the three isolates characterized as “inconclusive” showed that isolate 19 was susceptible in 72% of valid tests by the “BD” method (13 susceptible versus 5 resistant results) and susceptible in 100% of valid tests by both the “A” and the “C” methods. This strain had a* pncA* His82Tyr mutation and no other drug resistance. Isolates 21 and 27 were found to be primarily resistant using the “BD” method (67% and 61%, resp.) and primarily susceptible by the “A” and “C” methods (72% and 78%, resp.). This isolate had a* pncA* Ala28Thr mutation and was also resistant to isoniazid (INH), rifampin (RMP), and ethambutol (EMB). The occurrence of “Timed-Out” events increased from 2 using the “BD” inoculum method to 10 and 17 using the “A” and “C” methods, respectively. Excluding 19 “Timed-Out” events that occurred in one strain (isolates 11 and 23), the “Timed-Out” totals become 1, 4, and 5 for the “BD,” “A,” and “C” methods, respectively. The median number of days from test initiation to completion (excluding “Timed-Out” events) was 9, 10, and 11 days for the “BD,” “A,” and “C” methods, respectively.

The diagnostic reliability of the three PZA susceptibility tests (“BD,” “A,” and “C” inoculation methods) is addressed in Table 3. This table presents the percentages for either resistant or susceptible results that were true-resistant or true-susceptible, respectively. For example, in the case of the “BD” method, there were 116 resistant results (52 true- and 64 false-resistant); therefore, 44.8% (52 of 116) of the resistant results were true-resistant. Likewise, for the “BD” method, 98.9% (186 of 188) of susceptible test results were true-susceptible. These percentages correspond to the positive and negative predictive values of the test. There was a distinct increase in the percentages of resistant results that were true-resistant: from 44.8% (52 of 116) for the “BD” test to 71.2% (42 of 59) and 84.0% (42 of 50) for the “A” and “C” tests, respectively. Conversely stated, the percentages of resistant results that were false-resistant declined from 55.2% (64 of 116) for the “BD” test to 28.8% (17 of 59) and 16.0% (8 of 42) for the “A” and “C” tests, respectively. In summary, the positive predicative value of a resistant test result was greatly improved using the two lower density inocula. There was a minimal difference between test methods in the percentages of susceptible results that were true-susceptible, all methods having values greater than 95%.

Inoculum concentration had little or no effect on the susceptibility results for the isolates in the “always susceptible” category (Table 4). Five of the isolates in the “always susceptible” category had no false-resistant results, irrespective of inoculum concentration (isolates 1, 8, 11, 22, and 23). One isolate (number 18) had four false-resistant results using the “BD” inoculum and none using the two reduced inoculum methods. In sharp contrast, inoculum concentration had a substantial impact on the PZA susceptibility results for those isolates categorized as “predominately susceptible” (isolates 12, 13, 16, 24, 25, and 26). There were far fewer false-resistant results using the two reduced inoculum methods as compared to the “BD” method. Considering the combined results of all 20 isolates, there was a statistically significant difference (Pearson's Chi-Square test, *p* value < 0.05) between the “BD” and “A” methods and the “BD” and “C” methods but not between the “A” and “C” methods. Chi-Square analysis of the three methods could not be validly performed for individual isolates due to the small number of tests conducted.

The study results and predicted MGIT PZA test results were compared to assess the accuracy of those results for each of the dilution methods (Table 5). An accurate result was one in which the actual experimental categorical result was the same as the predicted result. Conversely, an inaccurate result occurred when the reported categorical result was contrary to the predicted result. Test accuracy improved from 78.3% (238 of 304) using the “BD” inoculum to 90.5% (268 of 296) and 94.1% (272 of 289) using the “A” and “C” inocula, respectively. While accuracy measured the correctness of the test results, precision measured the reproducibility of the results regardless of accuracy (Table 6). The percentage of precise and accurate results increased from 72.2% (109 of 151) using the “BD” inoculum to 87.6% (127 of 145) and 93.5% (131 of 140) using the “A” and “C” inoculum dilutions, respectively. A corresponding reduction in the number of precise but inaccurate results occurred, declining from 23 instances for the “BD” inoculum method to 10 for the “A” method and 4 for the “C” method. The percentage of imprecise results declined from 12.6% (19 of 151) using “BD” inoculum method to 5.5% (8 of 145) and 3.6% (5 of 140) using the “A” and “C” inocula methods, respectively. Overall, both test accuracy and precision were markedly improved using the two reduced cell density inocula as compared to the “BD” inoculum.

## 4. Discussion

The results of this study provide compelling evidence that the cell density of the inoculum used in MGIT PZA susceptibility testing can have a profound impact on test accuracy. This inoculum effect does not appear to be a universal phenomenon but rather seems to be limited to a subset of strains. When PZA susceptibility test results for each inoculum method were examined individually, there was a clear distinction between those isolates classified as “always susceptible” to 100 *μ*g/ml PZA and those in the “predominately susceptible” category. The six “predominately susceptible” isolates had a high degree of false resistance when the “BD” method was used; however, all produced significantly fewer false-resistant results when a lower inoculum density was used. In contrast, there were few false-resistant results among the “always susceptible” isolates, regardless of inoculum concentration. Some strains are clearly very responsive to inoculum effect, and for those strains, using methods with a lower cell density inoculum greatly decreased the likelihood of a false-resistant result. Of the two strains that were deemed “inconclusive,” study results indicate that isolate 19 could most likely be categorized as susceptible, because all sites found it to be susceptible using the “A” and “C” methods. The other “inconclusive” strain (isolates 21 and 27), while showing reduced resistance using the “A” and “C” methods, did not demonstrate clear susceptibility to PZA and therefore its true categorization remains inconclusive.

This study focused on PZA false resistance; therefore, only three strains predicted to be resistant were included. Two of these strains (numbers 4 and 14) had PZA MICs well above the critical concentration of 100 *μ*g/ml and were included in the study as resistant controls. These two strains were PZA-resistant across all laboratories and inoculation methods with the exception of one laboratory that had four false-susceptible results for one strain (number 14) using the “A” and “C” methods. Since these findings were limited to a single laboratory, we postulate that these were erroneous results. The third PZA-resistant strain (number 17) was multidrug-resistant and was involved in several TB outbreaks in the early 1990s [29–31]. There are conflicting reports concerning the PZA sensitivity of this strain [32, 33]. This inconsistency is theorized to result from the Thr47→Ala* pncA* mutation producing a PZA MIC near the critical concentration [34]. Based on prior MGIT PZA testing, using the standard (“BD”) protocol, we categorized this strain as “predominately resistant,” and the study results corroborate our experience. When tested using the lower cell density “A” and “C” inocula, the numbers of susceptible and resistant results were nearly equivalent. This strain was purposefully chosen for the study to demonstrate that achieving an accurate MGIT PZA sensitivity test result can become even more challenging when the MIC of the strain is near the critical concentration [35]. In such instances, the result obtained is likely to be highly affected by inoculum density.

An important concern regarding the use of a lower cell density inoculum is the impact on the length of time required to complete the test. An inverse relationship between the cell density of the inoculum and the time to test completion was anticipated. Just such a relationship was observed, with the time to test completion being extended either one or two days using the intermediate and lowest cell density inocula, respectively. A one- or two-day delay in test completion in exchange for a significant reduction in false-resistant results—and concomitant improvement in test accuracy—may be acceptable. In some instances, however, using a reduced cell density inoculum could result in failure to reach test completion within the 21 days allotted by the instrument protocol. An increase in the number of such “Timed-Out” events using the lower cell density inocula was observed in one strain (isolates 11 and 23). This strain, known to be especially slow growing, was selected for this study based on that characteristic. In our experience, such languid strains are uncommon. Excluding this unusual strain, there was only a minor increase in the occurrence of “Timed-Out” events using the lower cell density inocula. The possibility of an incomplete test while using a more dilute inoculum may be acceptable given the substantial reduction in false-resistant results observed in this study.

There are numerous possibilities regarding how these findings can be translated into laboratory practice. One option would be to initially test all isolates using the “C” method inoculum and then scrutinize each result taking into consideration the isolate's antibiogram. We have shown that a PZA-resistant result using the lower cell density “C” inoculum has a much higher positive predictive value than a resistant result using the standard (“BD”) inoculum. When an isolate is found PZA-resistant using the “C” method and is also resistant to one or more of other first-line antituberculosis drug(s), the verity of that PZA result is bolstered, since PZA monoresistance in* M. tuberculosis* is quite rare [36]. A PZA-resistant result, in combination with no other first-line drug resistance, could still be erroneous using the “C” method, as false resistance was not eliminated although it was significantly reduced. Another explanation for a PZA monoresistant result is that the isolate is* M. bovis* (inherently PZA-resistant due to a specific* pncA* polymorphism) rather than* M. tuberculosis*. The dilemma of a PZA monoresistant result would be encountered far less frequently using a reduced cell density inoculum.

We found little evidence that a lower cell density inoculum increases the occurrence of false-susceptible results, with the exception of one isolate whose PZA MIC was near the critical concentration. Since PZA-resistant* M. tuberculosis* is typically resistant to one or more of other antituberculosis drugs, the likelihood of a false-susceptible PZA result is quite low when the isolate is pan-susceptible. In the situation where an isolate is found PZA-susceptible using a lower cell density inoculum but has other first-line drug resistance, it might be prudent to retest that isolate using the standard inoculum method to preclude the remote possibility of false susceptibility. Overall, using a reduced cell density inoculum might result in far less need for repeat PZA testing.

## 5. Conclusions

This study clearly demonstrates that MGIT PZA sensitivity testing is subject to false-resistant results and that this problem can be mitigated using a lower cell density inoculum. While this problem and the proposed solution have been previously reported, those studies were limited to a single laboratory [25, 37]. This was a highly controlled study involving multiple laboratories, all testing the same panel of strains, using identical methods. This study establishes proof of concept that using a reduced cell density inoculum can improve the accuracy of MGIT PZA sensitivity testing by reducing false-resistant results. These findings provide the scientific basis that may inform a laboratory's decision to consider implementation of a modified MGIT PZA sensitivity testing protocol. Clinical laboratories would need to validate the modified method according to appropriate regulatory requirements prior to implementation.

## Figures and Tables

**Table 1:** Characteristics of 20 *M. tuberculosis *strains used in the multicenter evaluation.

Isolate number (duplicate)	Susceptibility to PZA (100 *µ*g/ml)			*pncA *mutation		Other drug resistance^b^
	(multiple tests)		PZA MIC^a^	Nucleotide	Amino	
	Consistently	Predominately	(ug/ml)	number	acid	
1	Susceptible	—	≤25	C 195 → T	Ser65Ser	CIP
4	Resistant	—	>800	A(-11) → G	NA^c^	RIF, CIP
7	Susceptible	—	50	None	NA	None
8	Susceptible	—	≤25	None	NA	None
11 (23)	Susceptible	—	≤25	None	NA	None
12 (24)	—	Susceptible	75	None	NA	None
13 (25)	—	Susceptible	75	None	NA	None
14	Resistant	—	>100	T 37 → C	Phe13Leu	INH
16 (26)	—	Susceptible	75	None	NA	FQ
17	—	Resistant	200	A 139 → G	Thr47Ala	INH, RIF, AMK, EMB
18	Susceptible	—	75	C 509 → T	Ala170Val	None
19	Inconclusive	Inconclusive	100	C 244 → T	His82Tyr	None
20	Susceptible	—	75	G 538 → A	Val180Ile	None
21 (27)	Inconclusive	Inconclusive	100	G 82 → A	Ala28Thr	INH, RIF, EMB
22	Susceptible	—	50	A 110 → T	Glu37Val	None

^a^Number of tests varied by isolate. When multiple tests were performed, the value is the approximate median. ^b^CIP: ciprofloxacin; RMP: rifampin; INH: isoniazid; AMK: amikacin; EMB: ethambutol. ^c^Regulatory mutation.

**Table 2:** Compiled PZA results for 14 susceptible and 3 resistant isolates^a^ tested in duplicate by 9 laboratories^b^.

Outcome	Predicted total	Number (%) by inoculation method		
		BD	A	C
True-susceptible	252	186 (61.2)	226 (76.4)	230 (79.2)
True-resistant	54	52 (17.1)	42 (14.2)	42 (14.5)
False-resistant	0	64 (21.1)	17 (5.7)	8 (2.8)
False-susceptible^c^	0	2 (0.7)	11 (3.7)	9 (3.1)

Total valid tests	306	304^d^	296^d^	289^d^
Timed Out^e^	0	2 (0.7)	10 (3.3)	17 (5.6)

Total tests^f^	306	306	306	306

^a^Results from three isolates categorized as “inconclusive” not included. ^b^Results for one laboratory excluded due to <70% reproducibility. ^c^18 of the false-susceptible results occurred in isolate 17. The remaining 4 occurred in isolate 14, all in the same laboratory. ^d^Denominators used to calculate outcome percentage. ^e^Test result not achieved within the 21-day instrument protocol. 19 of these events occurred in one strain (isolates 11 and 23). ^f^Denominator used to calculate Timed-Out percentages.

**Table 3:** Summary of categorical MGIT PZA susceptibility test results stratified by possible outcome and inoculation method.

Inoculation method	Susceptibility test result							
	Resistant				Susceptible			
	Number/category		Percent of total		Number/category		Percent of total	
	True	False	True^a^	False	True	False	True^b^	False
BD	52	64	44.8	55.2	186	2^c^	98.9	1.1
A	42	17	71.2	28.8	226	11^c^	95.4	4.6
C	42	8	84.0	16.0	230	9^c^	96.2	3.8

^a^Percentages correspond to the positive predictive value of a resistant result being true-resistant. ^b^Percentages correspond to the negative predictive value of a susceptible result being true-susceptible. ^c^18 of the false-susceptible results occurred in isolate 17. The remaining 4 occurred in isolate 14, all in the same laboratory.

**Table 4:** Comparison of predicted to actual MGIT PZA susceptibility test results stratified by inoculation method. Cumulative results of 9 laboratories^a^ each testing 20 isolates in duplicate.

Isolate number^b^	Predicted result^c^ (category)	Actual number of results by inoculation method^d^					
		BD		A		C	
		S	R	S	R	S	R
1	S (A)	18	0	18	0	18	0
4	R (A)	0	18	0	18	0	18
7	S (A)	17	1	17	0	16	1
8	S (A)	18	0	18	0	18	0
11	S (A)	18	0	14	0	12	0
12	S (P)	1	16	13	5	15	3
13	S (P)	12	6	18	0	17	0
14	R (A)	0	18	2^e^	15	2^e^	16
16	S (P)	9	9	15	2	18	0
17	R (P)	2	16	9	9	7	8
18	S (A)	14	4	18	0	18	0
19	INC	13	5	18	0	18	0
20	S (A)	17	1	18	0	18	0
21	INC	6	12	13	5	14	4
22	S (A)	18	0	18	0	18	0
23	S (A)	17	0	16	0	12	0
24	S (P)	3	15	11	7	15	3
25	S (P)	12	6	18	0	17	0
26	S (P)	12	6	15	3	17	1
27	INC	7	11	14	4	13	5

^a^Results for one laboratory excluded due to <70% reproducibility. ^b^Isolate numbers correspond to those in Table 1. ^c^S: susceptible; R: resistant; INC: inconclusive; A: always; P: predominately. ^d^Total results by method less than 18 (9 laboratories × 2 tests per isolate) indicate that some tests “Timed-Out” prior to a result. ^e^All 4 false-susceptible results occurred in the same laboratory.

**Table 5:** Accuracy of MGIT PZA susceptibility test results stratified by inoculation method.

Accuracy	Percentage (number) by inoculation method		
	BD	A	C
Accurate^a^	78.3 (238)	90.5 (268)	94.1 (272)
Inaccurate^b^	21.7 (66)	9.5 (28)	5.9 (17)

Total^c^	304	296	289

^a^Actual and predicted categorical results were the same. ^b^Actual and predicted categorical results were different. ^c^Denominators used to calculate accuracy percentages.

**Table 6:** Precision of MGIT PZA susceptibility test results stratified by inoculation method. Reproducibility of categorical results for each duplicate pair of isolates.

Precision	Percentage (number) by inoculation method		
	BD	A	C
Precise^a^ & accurate	72.2 (109)	87.6 (127)	93.5 (131)
Precise & inaccurate	15.2 (23)	6.9 (10)	2.9 (4)
Not precise^b^	12.6 (19)	5.5 (8)	3.6 (5)

Subtotal^c^	151	145	140
<2 results^d^	1.3 (2)	5.2 (8)	8.5 (13)

Total^e^	153	153	153

^a^Each duplicate isolate had the same categorical susceptibility result. ^b^Each duplicate isolate had a different categorical susceptibility result. ^c^Denominators used to calculate precision percentages. ^d^Time-Out occurred in one or both of the duplicate pairs of isolates. ^e^Denominator used to calculate <2 results percentages.
